# Recommendations for sharing network data and materials

**DOI:** 10.1017/nws.2024.16

**Published:** 2024-10-30

**Authors:** Zachary P. Neal, Zack W. Almquist, James Bagrow, Aaron Clauset, Jana Diesner, Emmanuel Lazega, Juniper Lovato, James Moody, Tiago P. Peixoto, Zachary Steinert-Threlkeld, Andreia Sofia Teixeira

**Affiliations:** 1 Michigan State University, East Lansing, USA; 2 University of Washington, Seattle, USA; 3 University of Vermont, Burlington, USA; 4 University of Colorado, Boulder, USA; 5 Santa Fe Institute, Santa Fe, USA; 6 Technical University of Munich, München, Germany; 7 University of Illinois, Urbana Champaign, IL, USA; 8 Institut d’Etudes Politiques de Paris, Sciences Po, Paris, France; 9 Duke University, Durham, USA; 10 Interdisciplinary Transformation University, Linz, Austria; 11 University of California, Los Angeles, USA; 12 Network Science Institute, Northeastern University, London, UK; 13 LASIGE, Faculdade de Ciências, Universidade de Lisboa, Lisboa, Portugal

**Keywords:** Ethics, data sharing, guidelines, open access, open data, open science

## Abstract

One of the goals of open science is to promote the transparency and accessibility of research. Sharing data and materials used in network research is critical to these goals. In this paper, we present recommendations for whether, what, when, and where network data and materials should be shared. We recommend that network data and materials should be shared, but access to or use of shared data and materials may be restricted if necessary to avoid harm or comply with regulations. Researchers should share the network data and materials necessary to reproduce reported results via a publicly accessible repository when an associated manuscript is published. To ensure the adoption of these recommendations, network journals should require sharing, and network associations and academic institutions should reward sharing.

## 1. Introduction

One of the goals of open science is to promote the transparency and accessibility of research. Sharing research data and materials is critical to these goals. However, the unique structure and the detailed information that networks contain can present challenges to sharing network data and materials. To help navigate these challenges, this article presents recommendations for sharing network data and materials that were developed by an intellectually, geographically, and demographically diverse working group at the request of the International Network for Social Network Analysis (INSNA).

Members of the working group were recruited from respondents to a survey about guidelines for reporting about network data (Neal, 2023b), with the goal of ensuring representation from multiple disciplines, regions, and demographic groups. We began by reviewing existing principles and expectations for data sharing, including the TOP Guidelines (transparency and openness promotion; Nosek et al., 2015), the FAIR data principles (findability, accessibility, interoperability, and reuse; Wilkinson et al., 2016), the CARE principles for Indigenous Data Governance (collective benefit, authority to control, responsibility, and ethics; Carroll et al., 2020), and the expectations of network journals. We then identified gaps in, or opportunities to clarify, these principles and expectations in the case of network data. Our goal was not to develop an entirely new set of recommendations but instead to review and adapt existing recommendations for their applicability to network research and then to formally endorse sharing data and materials in this domain. In this article, we present our detailed recommendations concerning whether, what, when, and where network data and materials should be shared. In general, *we recommend that network data and materials should be shared*, but access and use may be subject to restrictions under certain circumstances.

We recognize that current data sharing practices vary widely among network researchers and their respective disciplines. Therefore, we offer these recommendations as a first step toward establishing norms and expectations for data sharing in network research. We also recognize that sharing data can be challenging, but we view it as just as important to the research process as data collection, analysis, and interpretation, which can also be challenging. Nonetheless, following these recommendations can often require institutional support, and a lack of institutional support may impose barriers to sharing data despite researchers’ best efforts. Therefore, in addition to recommendations for researchers, we also discuss the role of institutions in supporting data sharing and offer recommendations for institutions to support researchers.

In section 2, we begin by defining key terms, reviewing guiding principles, discussing reasons to share data, and network-focused journals’ current expectations for sharing data and materials. In section 3, we present recommendations for whether, what, when, and where network data and materials should be shared. In section 4, we address potential concerns about sharing network data and materials. In section 5, we discuss the role of institutions in supporting researchers’ willingness and ability to follow these recommendations. Finally, in section 6, we summarize our recommendations for both researchers and institutions.

## 2. Background

### 2.1 Definitions

In these recommendations, we define a *network* as a set of nodes and the set of edges connecting them. We intend these recommendations to apply to all types of networks, regardless of what the nodes or edges may represent. We also intend these recommendations to apply in cases where the nodes and edges do not represent anything and where the object is an abstract mathematical object (i.e., a graph).

We define *data* as the information needed to reach the conclusions in a scientific manuscript dealing with network topics. We use this term broadly to include both empirical and simulated data, both qualitative and quantitative data, and both structural (e.g., the network itself) and nonstructural (e.g., node attributes) data.

We define *materials* as the non-data objects that are necessary to understand the data (e.g., documentation), that were used to collect the data (e.g., surveys, interview guides), and that were used to analyze the data (e.g., computer code, qualitative codebooks). In some cases, these materials are referred to as “meta-data.”

Finally, we define *sharing* as the noncommercial distribution of data and materials for use by others.

### 2.2 Guiding principles

To ensure that our recommendations for sharing network data and materials are consistent with broader norms of science, we reviewed several sets of guiding principles related to open science in general and data management in particular. While we do not necessarily endorse each of these principles, they informed the development of our recommendations.

The TOP Guidelines (Nosek et al., 2015) address a range of issues, including citation standards and pre-registration. With respect to sharing data and materials, they specify three increasingly stringent levels of transparency and openness:

1. a statement about the availability of data and materials;
2. data and materials are shared; and
3. data and materials are peer-reviewed.

These guidelines have been widely adopted, at different levels, by scientific authorities like the American Association for the Advancement of Science (AAAS) as well as hundreds of individual journals (McNutt, 2016). We aimed to develop recommendations consistent with the middle level.

The FAIR principles (Wilkinson et al., 2016) specify four characteristics that shared data and materials should have:

1. Shared data should be *Findable*, which means that they are stored in a searchable repository and have a unique identifier such as a Digital Object Identifier (DOI).
2. Shared data should be *Accessible*, which means that they are stored in a repository that is open and free to access.
3. Shared data should be *Interoperable*, which means they are stored in a standardized format.
4. Shared data should be *Reusable*, which means they are accompanied by detailed documentation and a license governing their use.

These principles have been widely adopted, including by the European Commission (European Commission, 2016). The FAIR principles informed the characteristics of shared network data and materials we sought to prioritize in our recommendations.

The CARE principles for Indigenous Data Governance (Carroll et al., 2020) are newer than TOP or FAIR and propose four requirements of shared data to ensure that the costs and benefits of sharing data are equitably distributed. Although they were originally framed in terms of the rights and interests of Indigenous people, they are relevant to protecting the interests of any subjects from whom data is obtained, particularly when they are members of a marginalized group:

1. Sharing data should provide *collective benefit*, which means that sharing should provide benefits not only to researchers but also to the subjects from whom the data was obtained.
2. The subjects from whom data was obtained should exercise *authority to control* shared data, which means that they can access the data and are involved in decisions about how it is maintained.
3. Researchers have a *responsibility* to cultivate respectful relationships with the subjects from whom data was obtained, including investing in the community’s capabilities to use these data.
4. Data sharing must follow *ethical* principles, including minimizing harm, maximizing benefits, and promoting justice.

The CARE principles informed our incorporation of ethics and equity in our recommendations, broadly ensuring that sharing network data and materials does not harm subjects.

### 2.3 Reasons to share network data and materials

Much has been written about the potential advantages of open data and data sharing (e.g., Huston et al., 2019; Murray-Rust, 2008; Piwowar and Vision, 2013). We believe that routine sharing of network data and materials offers the benefits of compliance, understanding, reproducibility, and efficiency. Thus, we are moving toward a “CURE” for restrictions on data that slow advances in network science.

First, the routine sharing of network data and materials will ensure that network research is in *compliance* with increasingly common mandates from public funding agencies that support network research. Since at least 2021, the European Commission has required grantees to “deposit the data in a trusted repository” (European Commission, 2021, p. 96), while starting in 2023, the US National Institutes of Health adopted an “expectation that researchers will maximize appropriate data sharing” (US National Institutes of Health, 2020).

Second, routinely sharing network data and materials associated with a published study facilitates readers’ *understanding* of the study. Although the methods section of an article typically provides a high-level summary of a study’s data (e.g., the definition of a node and an edge) and description of the analysis (e.g., what type of model), it may not provide fine-grained details such as a variable’s exact distribution or a model’s exact specification. Shared data and materials allow interested readers and fellow researchers to find these details, allow students to learn how to use the methods, and generally increase the understandability of the work and its scientific implications.

Third, shared network data and materials are essential for verifying the *reproducibility* of a study’s findings. Peer reviewers and readers who wish to verify a study’s findings can only do so if they have access to the underlying data and materials, which allow the findings to be independently reproduced. Moreover, reproducibility is the bedrock of extending research in new or deeper directions.

Finally, sharing network data and materials promotes the *efficiency* of network research. Network data can be resource-intensive to collect, and network surveys or network analytic code can be time-consuming to write. Sharing these data and materials with other network researchers can maximize their long-term benefits by reducing the effort necessary for future studies, while also facilitating the formation of collaborative teams working on common data or with common materials.

### 2.4 Current expectations

To understand the current expectations of network scholars, we reviewed the data-sharing policies of journals that frequently publish network research. Table 1 summarizes the expectations of eleven (families of) journals for a data availability statement and for sharing data and materials in May 2024. As the table illustrates, there is substantial variation in these expectations. This variation may be related to these journals’ histories, the primary disciplines they serve, or their publishers. In an interdisciplinary field where a network researcher might consider any of these journals as a possible outlet for their work or where they may find network research of interest, this variation in expectations may have the unintended consequence of establishing a hierarchy of transparency and rigor. The recommended practices for sharing network data and materials we propose below are intended to bring consistency to the expectations for transparency and rigor in network research.

## 3. Recommendations for sharing network data and materials

In this section, we offer recommendations for sharing network data and materials. These recommendations are organized around the questions network researchers confront when considering sharing data and materials: *whether*, *what*, *when*, and *where*.

### 3.1 Whether to share network data and materials

In view of the many benefits of sharing data and materials we discuss in section 2.3, we offer a broad recommendation: *network data and materials should be shared*. The question most researchers must confront is not whether to share the network data and materials associated with a publication, but whether those data and materials should be shared with or without restrictions. When there is no risk of harm and there are no applicable regulations, network data and materials should be shared without restrictions. In contrast, when it is necessary to avoid harm or to satisfy regulations, restrictions may be imposed on who can access shared data and materials or how shared data and materials can be used. In cases where the data is controlled by a third party, materials should be shared with instructions on how to access the data. In this section, we focus on when and what restrictions may be appropriate.

#### 3.1.1 When to restrict shared network data

For network data derived from human subjects, the CARE principles hold that researchers have an ethical obligation to protect the confidentiality and well-being of their research participants when sharing these data. The specific regulations that govern who or what is protected, and what protections they are afforded, vary by institution and jurisdiction. However, the broadly accepted Belmont Report (National Commission for the Protection of Human Subjects of Biomedical and Behavioral Research, 1979) articulated the principles of respect for persons, beneficence, and justice. The principle of respect for persons often involves obtaining informed consent from research participants. When informed consent is obtained, researchers should avoid providing broad assurances that “data will not be shared,” which may unnecessarily restrict sharing, and instead should explain when and with whom data may be shared (e.g., after de-identification, with peer reviewers). The principle of beneficence requires that sharing data benefits, or at least does not harm, research participants. When sharing data risks harming participants, consistent with the CARE principles, researchers may restrict access to minimize these risks.

Researchers also have an obligation to follow relevant laws, licenses, data use agreements (DUA), and other regulations. Such regulations often explicitly specify any restrictions that must be imposed on shared data. For example, data that carries a Creative Commons Attribution-NonCommercial (CC-BY NC) license may be shared, but with the restriction that it cannot be used for commercial purposes. In the case of data obtained from web-based sources, the terms-of-service or Application Programming Interfaces (API) documentation may specify not only how data may be collected from the source but also any restrictions on sharing that data (Fiesler et al., 2020).

Both ethical principles and laws exist to protect privacy. However, some unique privacy concerns exist when sharing network data. First, when some nodes are identifiable, it can be possible to make inferences about the identity or characteristics of other nodes (e.g., Jernigan and Mistree, 2009), including about nodes who represent individuals that did not directly consent to participate in the research and appear only as ‘secondary participants’ (Harris, 2008). Second, when the network system is identifiable, the network can provide a complete view of the system that otherwise would not be available and that could be exploited to cause harm. Third, even when the identities of the nodes are not provided, they can sometimes be re-identified through triangulation with other public data (Narayanan and Shmatikov, 2006). Although the risk of re-identification through triangulation is not unique to networks and can occur in any data, network data provide particularly rich information that may facilitate re-identification. When sharing network data carries a risk of potential harm, even if no human subjects ethical principles or regulations are applicable, and regardless of the original source of the data, it is still appropriate to impose restrictions on shared data to minimize those risks.

Ethical principles, legal regulations, and other sources of potential harm can justify imposing restrictions on shared network data and materials. However, there are limits to these justifications. First, they may justify restricting access to network data in which private individuals are identifiable but may not justify restricting access to network data that has been sufficiently de-identified (e.g., by removing identifying information) or fully anonymized (e.g., by destroying identifying information). For example, public-use AddHealth data is shared without restrictions on the Inter-university Consortium for Political and Social Research (ICPSR) repository. Second, they may justify restrictions on shared data, but not on shared materials. Because sharing research materials often presents fewer risks than sharing data, materials can often be shared without restrictions, even if they document sensitive data whose access requires restrictions. For example, although there are restrictions on accessing AddHealth network data, the associated materials (e.g., codebook, questionnaire) are shared without restrictions.

#### 3.1.2 What restrictions to impose on shared network data

When it is appropriate to impose restrictions on shared network data and materials, those restrictions may limit who can access the data and materials, how the data and materials can be used, or both.

First, access may be restricted to certain individuals. In some cases, this restriction may simply require users to provide contact information, which allows the data owner to monitor data use and provide updates about data corrections. In other cases, this restriction may require users to obtain authorization and be subject to oversight by a third party, such as an Institutional Review Board (IRB).

Second, access may be restricted to using the data and materials for certain purposes or may explicitly prohibit certain uses. Restrictions on how shared data can be used are typically defined by a license or DUA, which users are required to accept as a condition of access. If access is restricted to individuals subject to IRB oversight, then DUAs may mirror IRB regulations, requiring that data use complies with human subjects’ regulations. If access is restricted due to privacy-related risks, then DUAs may prohibit attempting to re-identify network nodes or re-sharing the data. In cases of highly sensitive data, DUAs may require that raw data are accessed only in controlled settings such as an approved data access center and that only approved aggregated findings are reported in publications.

The type of restrictions that are appropriate to impose on shared network data and materials are highly context dependent. However, regardless of the types of restrictions that are imposed, the need for, and nature of, restrictions must be clearly explained. For example, rather than indicate that ‘data are available upon suitable request,’ the researcher should explain why access is restricted and what conditions must be satisfied to grant access. Similarly, the restrictions should also be narrowly defined to minimize potential harms. For example, while it may be appropriate to prohibit disclosure of information that could be used to re-identify participants, it may not be appropriate to prohibit disclosure of all new findings derived from shared data.

#### 3.1.3 Inappropriate reasons not to share

There are several reasons that it may be appropriate to impose restrictions on shared network data and materials. However, it is generally inappropriate not to share data and materials, even with restrictions.

First, network data and materials should be shared even if the author intends to continue using it in future publications. However, we explain in section 3.2, it is only necessary to share the specific data and materials used to reach the results reported in a specific publication, which may represent only a subset of data from a larger project.

Second, network data and materials should be shared even if a journal does not require it. However, as Table 1 highlights, network journals increasingly do require sharing, and in section 5, we recommend that all network journals adopt such a requirement.

Third, network data and materials should be shared even if preparing these items for sharing requires additional time. The effort spent to fully document data and materials and prepare them for sharing is a key part of the research process. Moreover, some of this work is already part of the research process (e.g., recording how variables are coded) or is required by IRB procedures (e.g., de-identifying data for analysis). In section 5, we recommend that professional associations and academic institutions reward the effort associated with sharing network data and materials.

Finally, network data and materials should be shared even if the author believes it is unlikely they would be used by others. As we discussed in section 2.3, re-use is one reason to share data and materials, but sharing also makes it easier for readers to understand what a study has done and for students to learn new methods.

#### 3.1.4 Appropriate reasons not to share

Although we recommend that network data and materials should be shared, there may still be cases where sharing is not possible and therefore is not recommended. The most common such case is when the data are controlled by a third party, and an individual researcher does not have permission to share the data. For example, network data from the National Longitudinal Study of Adolescent to Adult Health (AddHealth) cannot be shared by the individual researchers who use it. In cases of third-party maintained data, researchers can satisfy the recommendation by sharing their materials and sharing instructions on how to access the data, even if the conditions of access are complex (e.g., an extensive application), impractical (e.g., a specific citizenship), or costly. In the AddHealth example, this might involve sharing the analytic code together with instructions on applying for access to the data via the Carolina Population Center’s Data Portal. Because other special cases may exist in which data cannot be shared, in section 5, we recommend that journal editors exercise discretion when applying expectations or requirements of sharing.

### 3.2 What to share

Once a researcher or research team decides to share their network data and materials, they must decide specifically *what* to share. To promote transparency, *we recommend that researchers share the network data and materials necessary to reproduce the findings reported in a given manuscript*. Optionally, researchers may choose to share additional data and materials that facilitate their reuse beyond simply reproducing the original findings.

A manuscript’s findings are reproducible if an independent researcher can obtain the same findings by analyzing the same data using the same methods as the original study. Achieving reproducibility requires sharing three items: the analytic subset of the data, the materials (e.g., documented computer code, qualitative codebook) used to analyze it, and a license. The analytic subset of the data refers to the portion of a potentially larger dataset that is analyzed and used to reach the findings reported in a given manuscript. For quantitative network data, this may include a subset of a larger collection of networks or a portion of a larger network, while for qualitative network data, this may include a portion of a longer interview or other document. While these data may represent only a subset of a larger dataset, they should be raw unprocessed data. For example, the shared data should include edge weights even if they are subsequently dichotomized to obtain an unweighted network and should include individual items even if they are aggregated into a scale. The format of the shared data may take whatever form is required to reproduce findings using the shared materials.

Researchers may choose to share additional data and materials that enable others to perform analyses that go beyond the original manuscript. When sharing for re-use, we encourage sharing data in a format that can unambiguously capture all necessary information and can easily be read using widely available and widely used software. For text-based data such as interviews, appropriate formats may include plaintext or, when additional text formatting is needed, markdown or rich text format. For quantitative network data, appropriate formats may include GraphML (Brandes et al., 2002) or graph modeling language (GML) (Himsolt, 1997), which are capable of recording the network’s structure, as well as node and edge attributes, and other meta-data. We also encourage sharing analytic materials that provide instructions about how to import the data and perform typical preprocessing and sharing detailed documentation about how, when, where, and why the data was collected (Bagrow and Ahn, 2022; Neal, 2023a; Luke et al., 2023). When possible, this documentation should include information about the missingness of nodes (e.g., are any nodes in the target population missing in the network) and uncertainty about edges (e.g., does a missing edge reflect evidence that it does not exist or a lack of evidence that it exists).

When preparing data for sharing, researchers should consider whether the data should be de-identified. Shared data should be de-identified if network members did not consent to being identified or if being able to identify the nodes or system could be used to cause harm. Many strategies for de-identifying network data exist: omitting node labels, omitting the identity of the system, omitting certain node attributes, coarsening node attributes by recoding into broader categories, jittering node attributes by adding random noise, and randomly adding or removing edges in a way that does not alter the network’s key topological properties. Researchers should carefully consider the effectiveness of these strategies because in some cases de-identified network data can be re-identified (e.g., Hay et al., 2008). Additionally, researchers sharing deidentified data should document the deidentification methods that were applied to the raw data.

Shared network data and materials should always be accompanied by a license that specifies how the shared data and materials can be used by others. Alternatively, when access to sensitive shared data and materials is restricted, the conditions of their access and use should be governed by a DUA. Licenses and DUAs should be as permissive as possible, imposing only those restrictions on access (e.g., agreement to maintain confidentiality) and use (e.g., crediting the creator with a citation) that are necessary. In cases where data are not sensitive or can be fully de-identified, “CC-BY” or “ODC-By” licenses that permit re-use with attribution may be appropriate. However, because licenses and DUAs are legal instruments, in complex cases, researchers may wish to consult a legal professional for advice.

### 3.3 When to share

After assembling the data and materials to be shared, researchers must decide *when* to share them. To ensure that readers can evaluate the evidence upon which research findings are based when those findings are officially disseminated, consistent with the TOP Level 2 guidelines, *we recommend that researchers share network data and materials when an associated manuscript is published*.

Researchers may choose to share their data and materials prior to publication. Specifically, consistent with the TOP Level 3 guidelines, researchers may share their data and materials privately with reviewers during the peer review process. There are challenges associated with sharing data and materials during the peer review process, including facilitating anonymous peer review and ensuring the data is not prematurely shared or re-used. For these reasons, although we do not formally recommend sharing data during peer review, we nonetheless encourage it.

### 3.4 Where to share

A final step in the process of sharing network data and materials requires deciding *where* to share them. To ensure accessibility and promote re-use, consistent with the FAIR guidelines, *we recommend that researchers share network data and materials in a repository that is publicly accessible, searchable versionable, and issues DOIs*.

This recommendation has five distinct components. First, we recommend that network data and materials are shared in a repository. In this context, a repository is a researcher-independent online platform for storing and disseminating files. As a researcher-independent platform, repositories offer greater accessibility and transparency than sharing data and materials on a researcher’s own website or offering to share them “on request,” which are insufficient.

Second, we recommend that the repository is publicly accessible. Public accessibility is important to ensure that other researchers can access the shared data and materials. Repositories that require paid accounts to access, or institutional repositories that are only accessible by members of the institution, are insufficient. While the repository may be publicly accessible, when they are sensitive, access to its contents may be governed by a DUA.

Third, we recommend that the repository is searchable. Searchability is important to ensure that other researchers can find shared data and materials. The repository should be searchable by the title of the dataset but ideally should also be searchable by author-specified keywords or by properties of the network (e.g., weighted, undirected).

Fourth, we recommend that the repository is versionable. Versioning refers to a repository’s ability to archive, with a timestamp, earlier versions of shared data and materials. It is important because it allows tracking the evolution of shared files, which may change as new data is added or as existing data is updated to correct errors. It can also mitigate the risk of researchers removing or un-sharing previously shared data.

Finally, we recommend that the repository issues DOIs. Having a DOI associated with a set of shared network data and materials is important because it makes them easier to find by providing a standardized hyperlink and makes them easier to cite because it provides a permanent identifier.

Table 2 lists some repositories that meet these recommended requirements and therefore are appropriate for sharing network data and materials. We offer no recommendation about choosing a specific repository. The choice of a repository may depend on several other factors highlighted in Table 2. First, researchers should choose a repository with longevity and stability; all of these repositories have existed for at least ten years. Second, researchers may need to consider the file size capacity of the repository; however, each of these repositories allows large files at no cost and even larger files by special arrangement. Third, if data and materials are shared for peer review at a journal that uses an anonymous review process, researchers should choose a repository that allows masking of the contributor’s identity, for example, by providing an anonymized link. Fourth, when sharing sensitive data for which access requires ethical or legal approval and completion of a DUA, researchers should choose a repository that offers restricted access options. Finally, to facilitate the discoverability of shared data and materials, researchers may want to choose the repository that is most widely used in a given discipline; for example, Harvard Dataverse is widely used in political science, while Open Science Foundation is widely used in psychology.

Repositories store network data and materials, making it accessible to other researchers. In addition to sharing network data and materials via a repository, researchers may also wish to have their shared network data included in an index, such as the Colorado Index of Complex Networks (ICON; Clauset et al., 2016) or Netzschleuder (Peixoto, 2020). Indexes do not store network data and therefore do not replace the need to share data via a repository. Instead, indexes provide a directory to network data stored in repositories, making them easier for researchers to find.

## 4. Potential concerns about sharing data

Open science practices such as data sharing date back many decades in some fields and journals, but are relatively newer and less familiar to others. As a result of this heterogeneity and the evolving norms around data sharing, researchers may have reasonable concerns about sharing data. In this section, we briefly address some possible concerns that sharing data and materials involves losses of time, data, privacy, and trust.

### 4.1 Time

Preparing data for sharing (e.g., de-identification, writing documentation) can involve a significant amount of time. Although sharing data adds an extra step to the research process, given the many benefits of sharing data we discuss in section 2.3, we believe this step is as important as the other steps in the research process (e.g., collecting data, analyzing data, interpreting data). Our hope is that these recommendations will lead network researchers to make sharing data a part of their typical research process and that adopting an intention to share at the beginning of a paper will minimize extra work when the paper is published.

### 4.2 Data

Collecting network data can require significant investments of time and money, from both the researcher and the research participants. After making such large investments in obtaining network data, researchers may be reluctant to simply give away their data, allowing it to be used by others who have not put in the hard work of data collection. However, sharing data is different from simply giving data away.

First, as we recommend in section 3.2, it is not necessary to share *all* network data and materials that have been collected. Instead, it is only necessary to share the specific pieces of data and associated materials that were used to reach the findings reported in a given manuscript and that are needed to reproduce those findings.

Second, when data and materials are shared, we recommend that they be shared with a license that specifies the conditions for their use. Ideally, data and materials are shared under permissive licenses that allow re-use without restriction, consistent with the FAIR guidelines. However, there are some reasonable restrictions that researchers may choose to impose on the conditions of reuse. One common family of licenses—Creative Commons or CC licenses—offers several such restrictions designated by two-letter codes. For instance, “CC-BY” requires that future users give credit to the creator, “CC-BY-SA” makes the additional requirement that future users share any adaptations of the data or materials, and “CC-BY-NC” additionally restricts re-use of the data and materials to noncommercial purposes. Another family of licenses—Open Data Commons—offers similar types, while being specifically tailored for data sharing instead of general copyrighted work. While we do not recommend their use, it is also possible for data to be shared with a highly restrictive license (e.g., a copyright), which permits the data to be viewed (e.g., to verify findings reported in a publication) but requires permission to re-use them.

Third, when data and materials are shared via a repository, they become a citable object similar to a published journal article. When others use the shared data or materials, a citation provides the creator credit for their effort in collecting and providing the data. Indeed, many journals have adopted Force11’s Joint Declaration of Data Citation Principles (Martone et al., 2014) to ensure that re-use of shared data is properly attributed.

### 4.3 Privacy

Network data is often particularly information-rich. They can provide a complete view of a system that otherwise would be invisible to individuals, can provide information about sensitive or private characteristics of research participants, and can enable inferences about the private characteristics of participants or even nonparticipants. Due to the risks of re-identifying individuals previously de-identified in network data or the risk of revealing private information of individuals (Tubaro et al., 2021; Horawalavithana et al., 2019), researchers may be reluctant to share network data. However, the risk of network data revealing private information must be balanced against the benefit of sharing data (see section 2.3). Such trade-offs must be considered on a case-by-case basis in the context of tools designed to mitigate these risks, including methods of de-identification and anonymization (e.g., Hay et al., 2008; Zhou et al., 2008) and restrictions that can be imposed on accessing the shared data and materials (see section 3.1.1).

### 4.4 Trust

Requiring researchers to share their data may be misinterpreted as an expectation or suspicion of fraud or a lack of trust in fellow scientists. While it is true that expectations of data sharing can reduce the incidence of academic fraud and make fraud easier to discover (Doorn et al., 2013; Chawinga and Zinn, 2019), these are not the primary motivations for a requirement to share. Rather than a practice for preventing bad academic behaviors, data sharing should be viewed as a practice for facilitating good academic behaviors, including being transparent, building on others’ work, helping the community, and helping students learn.

## 5. The role of institutions

These recommendations focus on steps that network researchers can take to make their work more transparent and open. However, researcher-focused recommendations are unlikely to be sufficient for promoting the transparency and openness of network research (Krähmer et al., 2023) because researchers’ willingness and ability to follow these recommendations occur within a broader institutional context. Promoting the transparency and openness of network research also requires that institutions both support network researchers in their efforts to share data and incentivize network researchers to do so. In this section, we discuss the role that three key institutions—journals, universities, and associations—play in this process, together with recommendations for how they can support and incentivize sharing of network data.

### 5.1 Journals

The role of journals, and specifically of editors and peer reviewers, is to judge whether the evidence supporting a researcher’s conclusions in a given article is sufficiently compelling to warrant publication. To ensure that this evidence is transparent to the rest of the scientific community and to allow readers to judge the evidence for themselves, *we recommend that journals require authors to follow these recommendations*. However, recognizing that special cases may arise where these recommendations are difficult to follow and that differences in institutional support may lead to differences in researchers’ ability to follow these recommendations, *we recommend that editors exercise discretion when enforcing this requirement*. Finally, because journal publications are a primary measure of researchers’ scholarly contributions and because shared data is an important scholarly contribution, *we recommend that journals review and publish submissions designed primarily to share and document data*.

### 5.2 Universities

The role of universities is to provide both support to, and recognition of, researchers. To support researchers’ efforts to share their data and materials, *we recommend that universities provide assistance with sharing data*, including help preparing data for sharing (e.g., de-identification, selection of license) and help drafting and implementing DUAs. To recognize the importance of sharing data, *we recommend that universities reward data sharing as a form of research productivity* in promotion, tenure, and other merit-based evaluations.

### 5.3 Associations

The role of professional associations is to promote and facilitate high-quality research within their respective fields and topics. To ensure that network research is transparent and open, *we recommend that associations encourage their members to follow these recommendations*. Additionally, to promote openness and transparency in network research, *we recommend that associations reward sharing data as a service to their respective communities*, for example, through awards for data sharing.

## 6. Conclusion

Table 3 summarizes our recommendations for sharing network data and materials. First, we recommend that *researchers should share their network data and materials* but may restrict access when necessary to prevent harm, comply with regulations, or protect privacy. Second, we recommend that *researchers should share the network data and materials necessary to reproduce reported results*, but researchers may choose to share additional data and materials that facilitate their reuse for other purposes. Third, we recommend that *researchers should share their network data and materials when an associated manuscript is published*, but researchers may choose to share earlier in the dissemination and peer review process. Finally, we recommend that *researchers should share their network data and materials in a repository that is publicly accessible, searchable, and versionable and offers DOIs*.

To support and incentivize network researchers to follow these guidelines, we also offer recommendations for key institutions. First, we recommend that journals require authors to follow these recommendations, but exercise discretion in enforcing this requirement. Second, we recommend that universities provide assistance with sharing data and reward sharing as a form of research productivity. Finally, we recommend that associations encourage their members to share data and reward sharing as a form of service.

## Figures and Tables

**Table 1.** Network journal data sharing expectations, retrieved May 1, 2024

Journal	Statement	Sharing
AAAS Journals (Science, Science Advances)	Required	Required
Applied Network Science	Required	No policy
Connections	No policy	No policy
Journal of Complex Networks	No policy	“Strongly encouraged”
Journal of Social Structure	No policy	No policy
NAS Journals (Proceedings, Nexus)	Required	Required
Network Science	Required	“Should…whenever possible”
Nature Journals (Complexity, Scientific Reports)	Required	Required
PLOS Journals (One, Complex Systems)	Required	Required
Social Networks	Encouraged	No policy
Social Network Analysis and Mining	Required	“Strongly encouraged”

(AAAS = American Association for the Advancement of Science; NAS = National Academy of Sciences; PLOS = Public Library of Science)

**Table 2.** Example repositories for network data and materials

Name	Established	Capacity		Masking	Restricted
		File	Project		Access
Figshare	2011	20 GB	20 GB	Yes	No
Harvard Dataverse	2007	2.5 GB	1TB	Yes	No
Inter-university Consortium for Political and Social Research (ICPSR)	1962	30 GB	30 GB	No	Yes
Open Science Foundation (OSF)	2013	5GB	50 GB	Yes	Yes
Zenodo	2013	50 GB	50 GB	No	Yes

**Table 3.** Recommendations for sharing network data and materials

	Recommendation
Whether	Network data and materials should be shared, but access may be restricted to prevent harm, comply with regulations, or protect privacy or may be restricted by a third-party
What	Data and materials necessary to *reproduce* the reported results
When	When an associated manuscript is *published*
Where	In a *repository* that is publicly accessible, searchable versionable, and issues DOIs
Journals	*Require* sharing network data and materials. Exercise *discretion* when enforcing this requirement. *Consider* submissions designed primarily to share and document data
Universities	Provide *assistance* with sharing network data and materials. *Reward* data sharing as a form of research productivity
Associations	*Encourage* members to share network data and materials. *Reward* data sharing as a form of service

## Data Availability

This paper does not report on data; however, the survey that was used to recruit the working group is described by Neal (2023b) and is available at https://osf.io/zjwm7/.
